# Effects of Warming on Chlorophyll Degradation and Carbohydrate Accumulation of Alpine Herbaceous Species during Plant Senescence on the Tibetan Plateau

**DOI:** 10.1371/journal.pone.0107874

**Published:** 2014-09-18

**Authors:** Changguang Shi, Geng Sun, Hongxuan Zhang, Bingxue Xiao, Bai Ze, Nannan Zhang, Ning Wu

**Affiliations:** 1 Key Laboratory of Mountain Ecological Restoration and Bioresource Utilization & Ecological Restoration Biodiversity Conservation Key Laboratory of Sichuan Province, Chengdu Institute of Biology, Chinese Academy of Sciences, Chengdu, China; 2 University of Chinese Academy of Sciences, Beijing, China; 3 Center for Ecological Studies, Sichuan Academy of Grassland Sciences, Chengdu, China; 4 Ecosystem Services, International Center for Integrated Mountain Development (ICIMOD), Kathmandu, Nepal; Tennessee State University, United States of America

## Abstract

Plant senescence is a critical life history process accompanied by chlorophyll degradation and has large implications for nutrient resorption and carbohydrate storage. Although photoperiod governs much of seasonal leaf senescence in many plant species, temperature has also been shown to modulate this process. Therefore, we hypothesized that climate warming would significantly impact the length of the plant growing season and ultimate productivity. To test this assumption, we measured the effects of simulated autumn climate warming paradigms on four native herbaceous species that represent distinct life forms of alpine meadow plants on the Tibetan Plateau. Conditions were simulated in open-top chambers (OTCs) and the effects on the degradation of chlorophyll, nitrogen (N) concentration in leaves and culms, total non-structural carbohydrate (TNC) in roots, growth and phenology were assessed during one year following treatment. The results showed that climate warming in autumn changed the senescence process only for perennials by slowing chlorophyll degradation at the beginning of senescence and accelerating it in the following phases. Warming also increased root TNC storage as a result of higher N concentrations retained in leaves; however, this effect was species dependent and did not alter the growing and flowering phenology in the following seasons. Our results indicated that autumn warming increases carbohydrate accumulation, not only by enhancing activities of photosynthetic enzymes (a mechanism proposed in previous studies), but also by affecting chlorophyll degradation and preferential allocation of resources to different plant compartments. The different responses to warming can be explained by inherently different growth and phenology patterns observed among the studied species. The results implied that warming leads to changes in the competitive balance among life forms, an effect that can subsequently shift vegetation distribution and species composition in communities.

## Introduction

Global warming has raised the global mean surface temperature by approximately 0.74°C over the period 1906–2005 and is predicted to further increase it by an additional 1.8–4.0°Cduring the 21st century [1]. This unprecedented warming has greatly impacted plant growth phenology and function across biomes, and attracted a growing alarm from ecologists [2]–[5]. Specially, one of the growing concerns of global warming is its potential effects on basic plant processes, such as the degradation of chlorophyll during senescence. Autumn plant senescence is a critical life process with adaptive significance for perennial plants because of its association with resorption of nutrients (especially nitrogen, which is considered to be a limit nutrient in terrestrial ecosystems) [6], [7] and carbohydrate storage in winter [8], [9]. One of the most fundamental processes linking plant N use and C storage, above and below ground, involves the seasonal degradation of chlorophyll. For example, some studies have reported that the photosynthetic activity of terrestrial vegetation increase in response to autumn warming [10], which affects the degradation of chlorophyll [11], [12] and therefore, the onset of senescence through N and C dynamics was also affected [12]–[14]. The potential effects of climate change on senescence and C usage are poorly understood provides the basis of our investigation.

The cessation of plant growth at the end of its growing season is mainly affected by air temperature and photoperiod [15], [16]. Thus, the timing of plant senescence is controlled by a trade-off between survival and productivity. For example, many trees track photoperiod and shed their leaves in autumn in order to protect them from frost damage (unfavorable conditions for photosynthesis) [17]. For some short-lived and early successional herbaceous species, temperature plays an important modulating role in controlling leaf senescence [3].

The Tibetan Plateau is one of the most sensitive areas to global climate change. During the period from 1955 to 1996, the average of regional temperature increased by 0.64°C, exceeding those in the Northern Hemisphere and on the same latitude zone during the same period [18]. A further increase of 0.6–0.9°C is projected for the period 2015–2050 over this region [19]. The vegetation of the Tibetan Plateau region is characterized as highly temperature-responsive [18]–[20], and so rapid changes of the prominent herbaceous communities in that region are expected to soon follow. Specifically, we predict that climate warming in autumn will reveal new functional groups defined by trade-offs between the conflicting requirements of N and C. Many studies have shown that the photosynthetic rate and terrestrial C sinks generally increase with the rise in temperature during growing seasons [21]–[24]. Reduced growth demands, in combination with a high photosynthesis capacity, likely results in the underground accumulation of more photosynthates, such as non-structural carbohydrates [25].

The significance of stored, non-structural carbohydrate for perennial plants has been widely documented in regard to early-spring growth [26], [27] and reproduction during the absence or shortage of photosynthesis [9], [16]. This suggests that the amount of carbohydrate storage in autumn will influence the plant growth and reproduction in the following growing season. Although significant effects to photosynthetic capacity, photosynthate allocation, and carbohydrate accumulation have been reported with elevated temperatures [23]–[25], [28], the response of root TNC to ongoing climate warming is unknown. In addition, patterns of plant senescence varies strongly between annuals and perennials [29]. Different species or functional types displayed different phenological sensitivities to temperature changes, which contributed to variations of the C balance [30]–[32] and the composition of ecosystem or community [33], [34]. However, little is known about the differences in the magnitudes of the response to autumnal warming among life forms. To contribute to an assessment of the impact of autumn warming on grassland ecosystems, we need to distinguish the different phenological responses across life forms in order to highlight potential alteration in ecosystem reconfiguration and species' competitive balance. Indeed, some species could enhance their competitive performances if they are able to rapidly improve their fitness under a warmer climate [30].

To assess the potential effects of isolated warming in autumn, we examined: chlorophyll dynamics; N status in leaves and culms; total non-structural carbohydrate (TNC) accumulation in roots; and the growth performances in the following growing season of four native herbaceous species. These species included *Elymus nutans*, *Delphinium caeruleum*, *Euphrasia regelii* and *Swertia mussotii*. The simulated warming field experiment was conducted in an alpine meadow on the Tibetan Plateau. Based on previous descriptions and the expected effects of warming in the region, we hypothesized that (i) autumn warming will slow down the degradation of chlorophyll in leaves and culms and promote higher N concentration in plant tissue, and the effect will vary among different life forms, and (ii) autumn warming will increase root carbohydrate accumulation for perennial species, which will promote plant growth performance and diversify plant phenological parameters in the following growing seasons.

## Study Site and Methods

### Study site and species

The study was conducted in the Hongyuan Alpine Meadow Ecosystem Research Station of the Chinese Academy of Sciences (32°48′N, 102°33′E and 3500m asl), located approximately 40 km to the north of Hongyuan county town, Sichuan Province, China. In the past 30 years (2011–1981), the mean annual air temperature for this area is 0.9°C with a mean monthly temperature ranging from −10.3°C in January to 10.9°C in July. Annual precipitation in this area is approximately 690 mm, with peak rainfall in May to August. The soil is classified as an alpine meadow soil according to the Chinese classification or Cryumbrept according to the US Soil Taxonomy. The study site was fenced in 2003, and prior to that, acted as pasture land planted with *Elymus nutans*. Currently, the predominant species are *Elymus nutans*, *Koeleria macrantha*, *Vicia unijuga*, and *Delphinium caeruleum*, which make up more than 80% of the total aboveground plant biomass of the meadow.

In this study, the species that were studied included two perennial species, *E. nutans* and *D. caeruleum,* and two non-perennial herbs, *Euphrasia regelii* and *Swertia mussotii*. The four species represent distinct life forms with a large natural distribution over the alpine meadow of the Tibetan Plateau. Among the four species, *E. nutans* is a caespitose grass species and its culms occur individually or in tufts that are either whole genets or ramets connected by rhizomes [35]. Each individual of *D. caeruleum* can bear multiple inflorescences. Both *E. regelii* and *S. mussotii* have short taproots. In particular, although *S. mussotii* is a monocarpic biennial species in this site, individuals selected are two-years old according to the reproductive organs [29]. The roots systems of the four herbaceous species are mainly distributed in 0–20 cm depth of soil. Flowering phenology varies from early July to early August, but seed formation occurs before late August.

### Individuals pre-selection and experimental set-up

We fenced in a 1 ha plot at the study site in mid-August, 2011 when the plants were fully mature. Within this plot, we selected and marked individual plants of the four selected species using consistent selection criteria. This criteria involved identification of individual plants of the same species with the same height, representing similar culms, and that were separated by more than 40 cm (in order to avoid clonal plants). The selected individuals were labeled with plastic cards, and two (usually the third and fourth fully expanded visible leaves from the top of the shoot for perennials) or eight (the third to tenth for non-perennials) leaves were marked on the petiole with red ink. The 1 ha plot was subdivided into control and warming treatment quadrates, which had 2 m * 2 m dimensions. Half of the individuals in each quadrate were randomly selected for the study in the first year, and the remaining individuals of *E. nutans* and *D. caeruleum* in the control and warming quadrates were also marked for the replicated study in the following year. Placing marker stakes near the shoot base, given the possibility the shoots could be blown away or decay, marked these individuals. In total, approximate 60 warming quadrates and 50 control quadrates were established in the natural field.

In the warming treated quadrates, we applied passive warming using a modified, larger version of the ITEX open-top chamber (OTC) [36]. Our OTCs were 80 cm high, and, in order to reduce the edge effects (such as precipitation), were 1.5 m wide at the top and 2 m at the base. Based on previous phenological observations, the autumn warming experiment was started on the 1st of September, 2011 before the onset of senescence. At completion of senescence, on the 4th of November, all the OTCs were removed from the warming quadrates and outlines of the quadrates were created.

**Figure 1:**
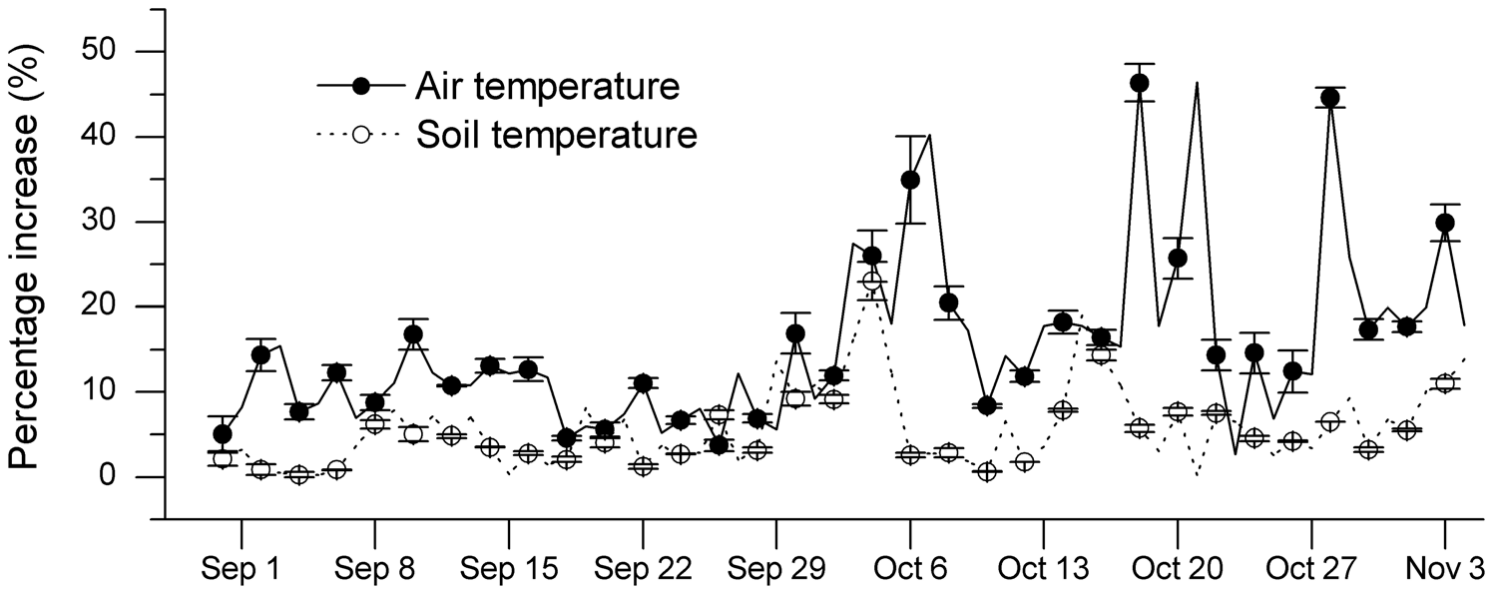
Effects of warming on air and soil temperature. Average percent increase in mean daily air temperature (25 cm above-ground, filled circles, solid line) and soil temperature (5 cm depth, open circles, dashed line) in warmig quadrates throughout the plant senescence process in 2011. Mean ± SD, n = 3.

HOBO Pro v2 series temperature (Onset Inc., Pocasset, Massachusetts) were placed in the center of quadrates to monitor the atmospheric temperature (25 cm above the ground) and the soil temperature (5 cm below the ground) at 30-minute intervals for control and warming quadrates during the warming period (Figure 1). Soil moisture levels (0–5 cm) were measured by a Diviner-2000 Portable Soil Moisture Probe (Sentek Pty Ltd., Balmain, NSW, Austrlia). Temperature and moisture were monitored in three quadrates for each treatment.

### Nitrogen in leaves and culms

The first collection of leaves and culms was carried out on August 20th, 2011 and the last collection was conducted when leaves were all senesced but still attached. Once the OTCs were established, collections were conducted at approximately 7-day intervals, and more frequently when sudden cooling or color change happened. For each species and each collection, three pre-selected individual plants (from 3 different quadrates for each treatment) were clipped. To minimize the effect of variation in culm size, 10-cm-long pieces of the culm base were used. For leaves, only blades were included, while both sheaths and stems were assigned to culms. After being oven dried at 65°C for 48 h, leaves and culms were weighed and ground in a ball mill (Retsch MM 400; Retsch GmbH & Co KG, Haan, Germany) for analysis later. The nitrogen concentration of each sample was analyzed using a Carlo Erba CN Analyzer (Milano Itay) and expressed in mg g^−1^.

### Chlorophyll determination

The chlorophyll concentration of intact leaves, representative of the leaf senescence process, was measured non-invasively by a SPAD-502 meter (Minolta Camera Co., Osaka) in 2011 between 10:00 and 16:00 h [37]. In addition, 32 leaves from individual species that ranged in the senescence process from green to senesced were collected from fields outside of the experimental quadrates and used to calibrate the SPAD-502. Separate measurements with the SPAD-502 were made on each leaf, and the average of the readings from all the selected leaves was given for the individual plant. Immediately after the SPAD-502 measurements, calibration leaf samples were collected, weighed and frozen in liquid nitrogen, and stored until chlorophyll extraction was performed in the laboratory. For chlorophyll extraction, the leaf samples were mechanically dissociated, suspended in 80% acetone and filtered. The extract absorbance was measured at 645, 663, and 720 nm using a spectrophotometer (UV-2401PC, Shimadzu Corp., Kyoto, Japan). The chlorophyll concentration was calculated and expressed relative to fresh leaf mass (mg g^−1^) [38]. Chlorophyll concentrations and SPAD-502 readings were used to create a non-linear standard curve allowing determination of chlorophyll concentration of experimental leaf samples using SPAD-502 readings.

### Non-structural carbohydrate

To estimate changes of non-structural carbohydrate concentrations, we harvested the root systems of individuals on the end phase of plants senescence (with the last collection of leaves and culms for all species) and before the emergence for perennials (10th of April 2012). For each treatment group, the root systems of 3 pre-selected plants for each species were chosen at random from 3 different quadrates and carefully collected using a custom-made root auger. After removing excess soil, the root samples were transported to the laboratory and stored overnight in a refrigerator. In the laboratory, roots were gently rinsed by hand, distinguished from roots of other species. It is important to note that it was impossible to collect the entire root system of any individual plant and as a consequence, the root biomass was slightly underestimated in this study. Root samples were ground using a mechanical ball for the analysis of TNC (total non-structural carbohydrate), which represents the sum of soluble sugars and starches. These were expressed in glucose equivalents and measured by colorimetric analysis. The sub-sample was sonicated in an acetate buffer for 3 minutes to further break up cells and then placed in a shaking water bath at 80°C for 1 hour with alpha-amylase added to solubilize amylopectins. After amyloglucosidase was added, the sample was shaken overnight at 60°C in water bath. A sub-sample of supernatant was removed and stored frozen until addition of phenol and sulfuric acid for colorimetric analysis with a spectrophotometer at 487 nm [39].

### Growth in 2012

In order to determine the potential effects of autumn warming, the growth performance and phenology of *E. nutans* and *D. caeruleum* were observed in the following growing season. All the pre-selected individuals that were not used during the plant senescence experiment were randomly separated into three groups, and used to assess the population phenology phases of each species. We tracked phenological progression in three to seven day intervals: from vegetation emergence to open flower, two key development stages of life history. The open flower stage was defined as the presence of open flowers or exposed stamens and/or styles for grasses. Each phenology phase was defined as the time when 10% of the species population was found to be in the phase lasting to the time when 90% of the population had entered the phase [40]. All phenological parameters were recorded as the number of days after January 1st, 2012.

Eight individuals of each species for each treatment were randomly selected from 3 different quadrates, harvested, dried at 80°C for 48 h, then weighed to assess the above-ground biomass of individuals. Harvest dates were May 15th (c. 2 weeks after the emergence) and August 17th (peak biomass), 2012.

### Statistical analysis

The effects of warming on N concentration in leaves and culms, chlorophyll content in leaves, TNC in roots, biomass of plants, and phenological parameters were analyzed using one-way ANOVA followed by a Tukey's post-hoc test for each collection of each species. Each group was tested for normality (Kolmogorov-Smirnov test) and log transformed to achieve the normal distributions when necessary. Relationships between chlorophyll content and SPAD-502 values and chlorophyll content and N concentration in leaves were explored using Pearson correlations. All statistical analyses were performed with SPSS 15.0 software for windows (SPSS Inc., Chicago, IL, USA).

## Results

### Effects of OTCs

We found our warming manipulations had consistently small but realistic effects on air and soil temperatures (Figure 1). Daily mean air temperature in the experimental quadrate was increased by 0.83°C compared to the control quadrates during the treatment period, and warming affected the daily mean soil temperature by 0.54°C. Data-loggers showed that soil moisture did not differ between warming and control sites (data not shown).

### Alteration of chlorophyll content during plant senescence

**Figure 2:**
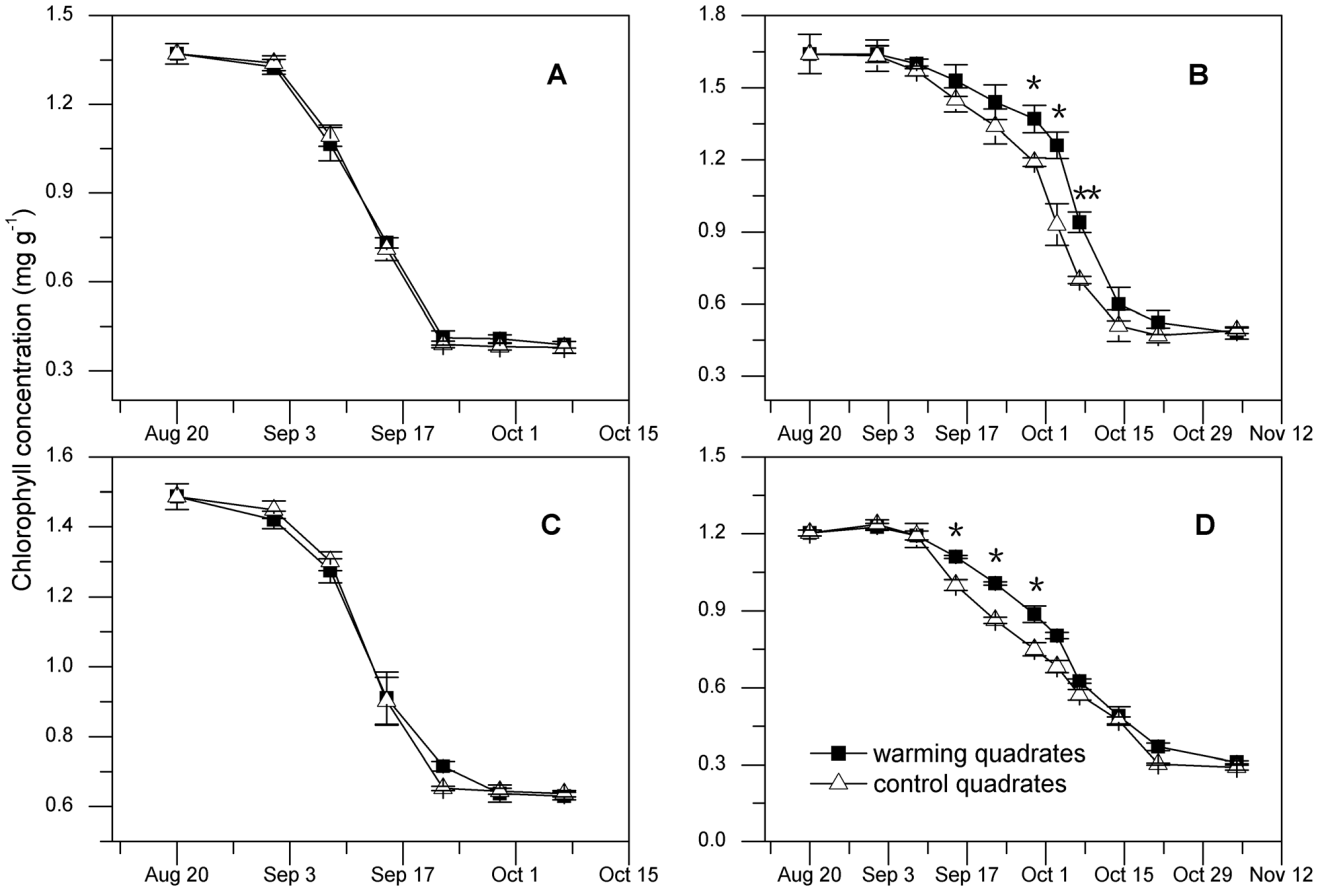
Effects of warming on chlorophyll concentration during the leaf senescence. Decline in chlorophyll concentration for E. *regelii* (**A**), *D. caeruleum* (**B**), *S. mussotii* (**C**) and *E. nutans* (**D**) in warming and control quadrates during the leaf senescence in 2011. Error bars are SE (n = 3). Asterisks indicate significant differences for each collection in warming and control quadrates: ** indicate *P*<0.01; * indicates *P*<0.05.

Timing of plant senescence phenology (decline in chlorophyll content) varied significantly among different plants, although the senescence occurred simultaneously on early September. Specifically, we found that non-perennial herbs (*E. regelii* and *S. mussotii*) ceased their senescence mainly by late-September, while perennials species (*E. nutans* and *D. caeruleum*) completed senescence by late-October (Figure 2). In general, the response of chlorophyll to autumn warming also varied among life forms: warming did not change the dynamics of chlorophyll for non-perennial herbs (Figure 2 A, C), while the decline of chlorophyll concentrations by warming in perennial plants was initially slowed but then accelerated in the later phase (Figure 2 B, D). Gradually, the chlorophyll concentration of those in the control treatment dropped significantly below that of warmed quadrates and the gap gradually increased with the duration of treatment. Consequently, chlorophyll concentrations of *D. caeruleum* (Figure 2B) and *E. nutans* (Figure 2D) in warming quadrates were significantly higher than that in controls. Despite all this, the effects of warming on the ultimate chlorophyll concentration in leaf litter were minor for all four species.

### Decline in N concentration in leaves and culms

**Figure 3:**
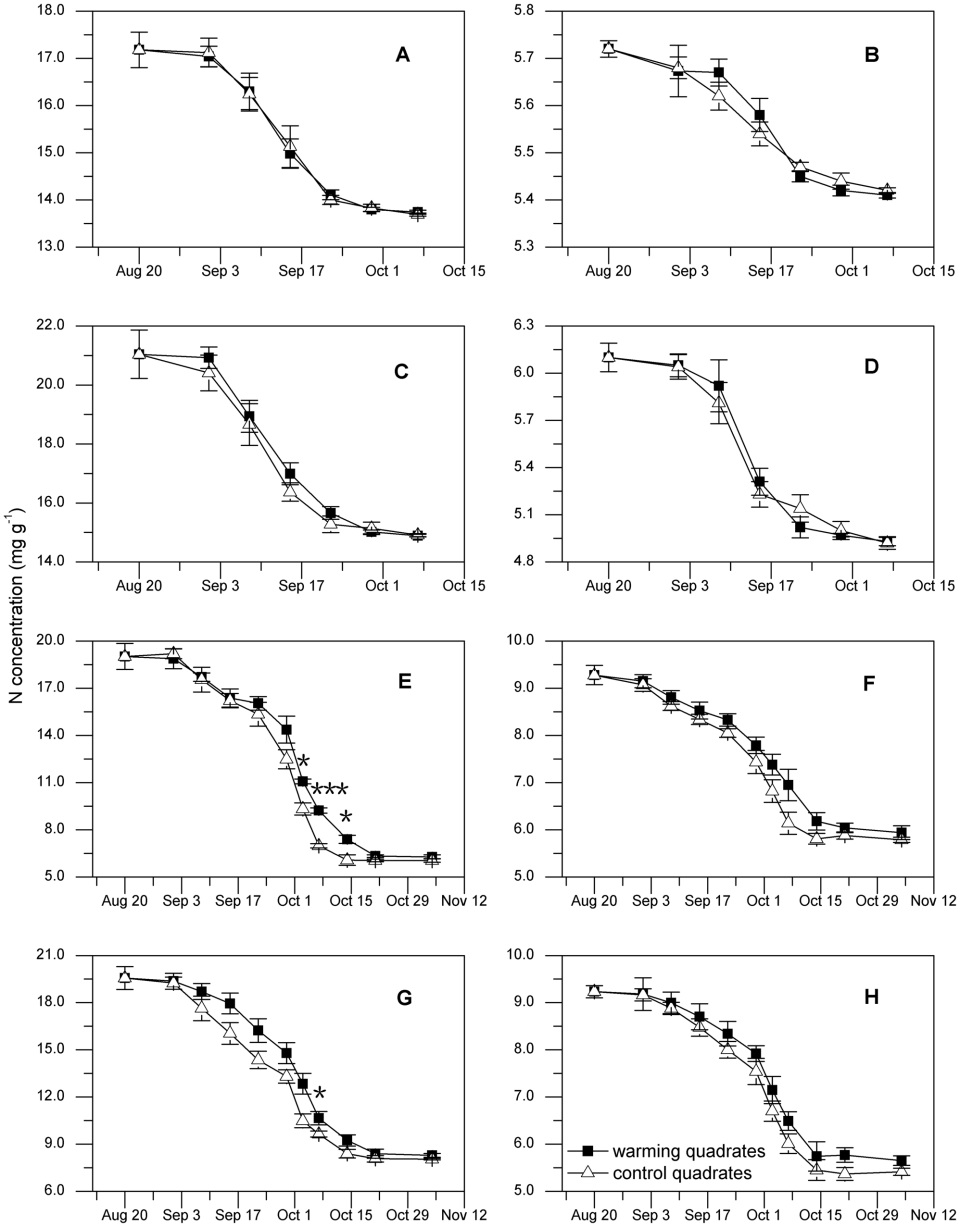
Effects of warming on N concentration during the plant senescence. N loss in leaves (the left column) and culms (the right column) for *E. regelii* (**A**, **B**), *S. mussotii* (**C**, **D**), *D. caeruleum* (**E**, **F**) *and E. nutans* (**G**, **H**) during the plant senescence in autumn, 2011. Error bars are SE (n = 3). Asterisks indicate significant differences for each collection in warming and control quadrates: *** indicate *P*<0.001; * indicates *P*<0.05.

There was an overall tendency for N concentration to decline during senescence, similar to the degradation of chlorophyll (Figure 2; Figure 3A, C, E, G). Declines in the N concentration of leaves for all plants started in early September and ceased earlier for *E. regelii* and *S. mussotii* than the perennial species, i.e. *D. caeruleum* and *E. nutans*. Warming did not change the N loss in leaves and culms of *E. regelii* and *S. mussotii*, while it did significantly slow the N loss in leaves of *D. caeruleum* and *E. nutans* during the senescence. In contrast to the positive response of leaf senescence, effect of autumnal warming on culms senescence of *D. caeruleum* and *E. nutans* was not found. Although the responses of senescence to warming varied significantly among species, the N concentration in the senesced tissues did not differ amongst the treatment groups for all species (Figure 3).

### Total non-structural carbohydrate (TNC) in roots

**Figure 4:**
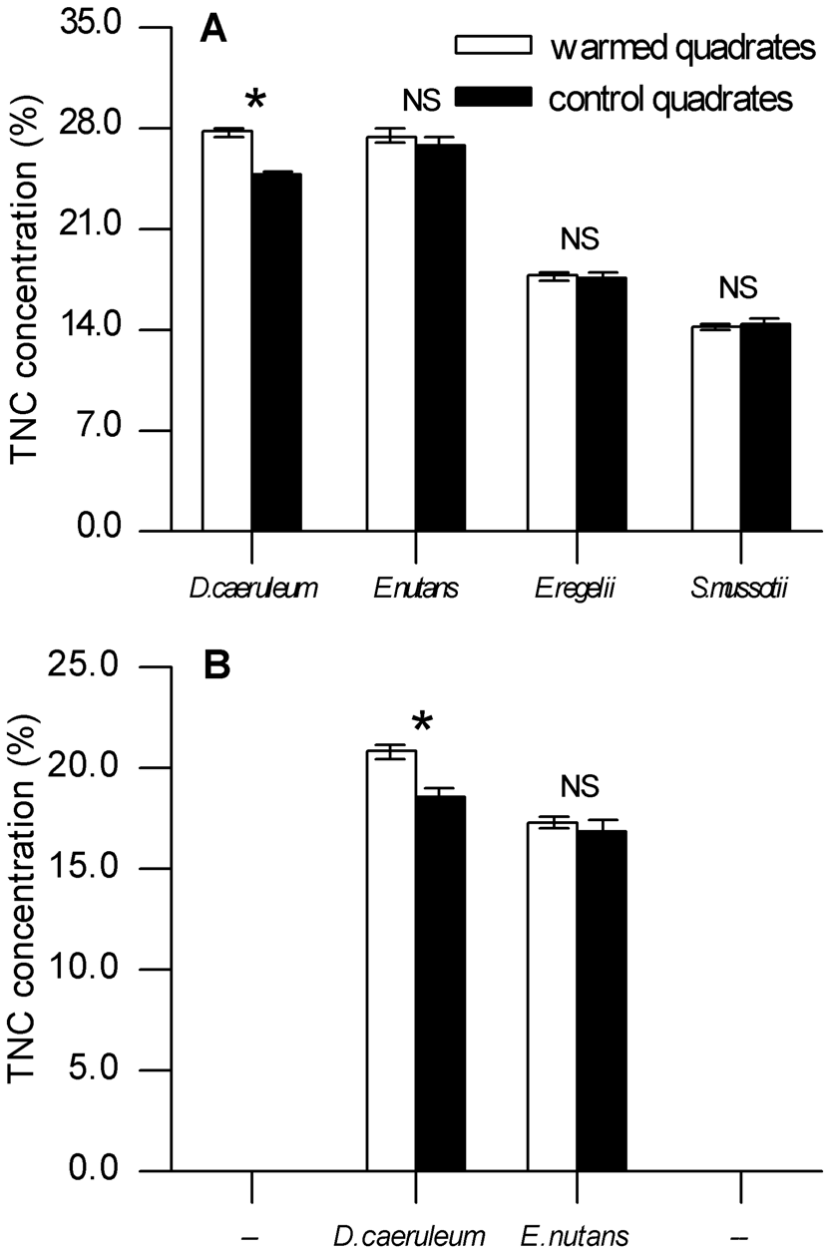
TNC (Total non-structural carbohydrate) concentration in roots. Accumulations of TNC measured (**A**, for *E. regelii*, *S. mussotii*, *D. caeruleum* and *E. nutans*) by the end of plant senescence in 2011 and (**B**, for *D. caeruleum* and *E. nutans*) in the beginning of spring in 2012. Error bars are SE (n = 3). Asterisks indicate significant differences (*P*<0.05) between warmed and control treaments. NS means not significant (*P*>0.05).

The responses of TNC accumulation to autumn warming were strongly species dependent (Figure 4A). For non-perennial species, i.e. *E. regelii* (*F*_1, 4_ = 0.073, *P* = 0.801) and *S. mussotii* (*F*_1, 4_ = 0.237, *P* = 0.652), root TNC was affected little by warmed air temperature. In contrast, we observed a significant increase of 11.83% in root TNC under warming temperatures for *D. caeruleum* (*F*_1, 4_  = 48.4, *P*  = 0.002; Figure 4A) by the end of plant senescence in 2011. While it is worthwhile noting that there was no significant alteration in root TNC found for *E. nutans* under warming temperatures (*F*_1, 4_ = 0.873, *P* = 0.403; Figure 4A). In early spring of 2012, root TNC differences between warming and control quadrates for the two perennial species were similar to that of 2011, although they were lower (Figure 4B). Moreover, the root TNC of *D. caeruleum* in warmed quadrates was still significantly higher than that in the control quadrates (*F*_1, 4_ = 15.31, *P* = 0.017).

### Growth performances in 2012

**Figure 5:**
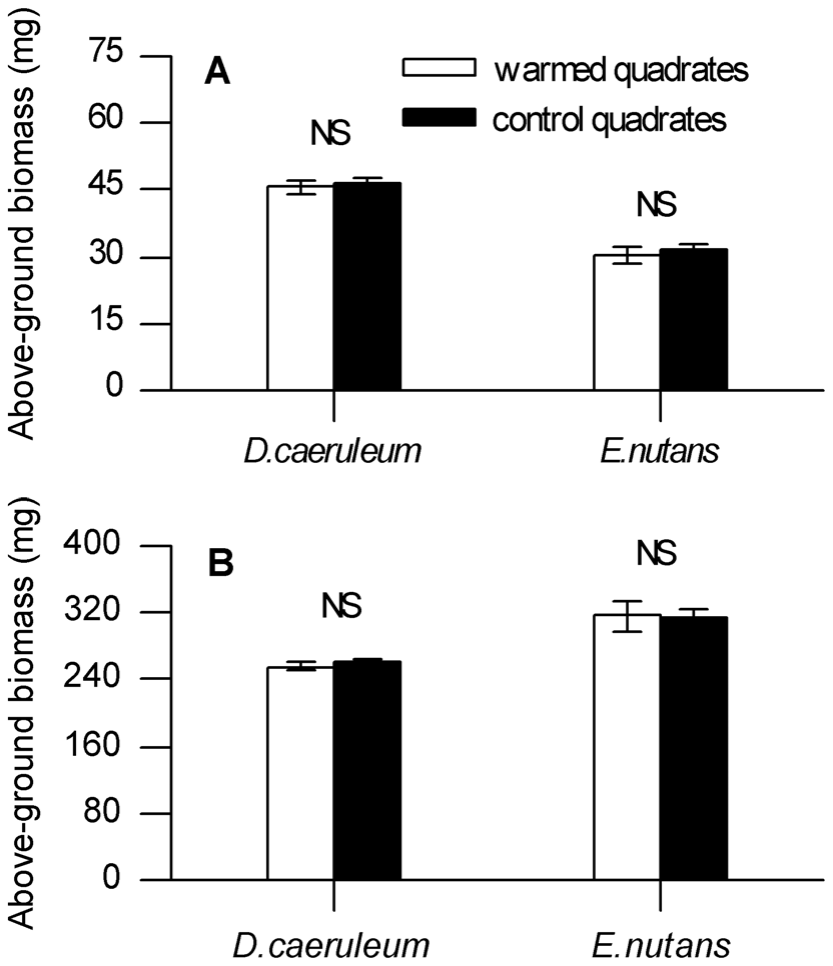
Individual plant above-ground biomass in 2012. Individual plant above-ground biomass for *D. caeruleum* and *E. nutans* (g, means ± SE, n = 8) measured (**A**) in the beginning of spring and (**B**) in late summer, 2012. Asterisks indicate significant differences (*P*<0.05) between warmed and control quadrates. NS means not significant (*P*>0.05).

Warming did not result in the above-ground biomass of individual plants deviated significantly from those in control quadrates for *D. caeruleum* and *E. nutans* not only in May but also in August, 2012 (Figure 5). Similarly, the phenological parameters preceding offspring production (emergence onset time, EOT; duration of emergence, DOE; open flower onset time, FOT; duration of open flower, DOF) did not differ between the warmed and control treatments for the two perennial herbaceous species (all *P* values> 0.05; Table 1).

**Table 1:** Phenological parameters for *D. caeruleum* and *E. nutans* in warmed and control quadrates.

Species	Phenological parameters	Warmed plots	Control plots	*P*
*D. caeruleum*	EOT	107.7±2.6	109.0±2.3	0.721
	DOE	22.0±1.5	22.7±1.8	0.789
	OOF	204.7±4.1	208.3±3.8	0.543
	DOF	48.7±3.2	46.3±2.6	0.601
*E. nutans*	EOT	97.7±3.8	96.7±3.3	0.853
	DOE	14.0±1.5	13.0±2.3	0.736
	OOF	207.3±3.5	208.0±4.4	0.911
	DOF	45.0±1.0	42.0±1.5	0.176

*Notes:* Means (±SE) of emergence onset time (EOT), duration of emergence (DOE), open flower onset time (FOT) and duration of open flower (DOF) for *D. caeruleum* and *E. nutans* in warmed and control quadrates conducted at an alpine meadow on the Tibetan Plateau. Means of phenological parameters are expressed as day of year (days after 1 January). The duration of emergence was calculated as the number of days from the emergence onset time to the emergence offset time; similarly, the duration of open flower is equal to the number of days from the open flower onset time to the open flower offset time. Significant differences between phenological parameters in warmed and control quadrates are denoted by *P* values.

## Discussion

### Effects of OTCs

Observational studies and warming experiments are two main approaches adopted by researchers to predict plant responses to global warming. They are suggested to be used in conjunction, since using only warming experiments may underpredict plant phenological responses to climate change [4]. One recent study found consistent result after comparison of experimental and observational results at single sites [41]. In our study, although the magnitude of warming was, on average, conservative and small compared with the recently reported warming manipulation [42]–[44], our study was not affected, since the sensitivity of plants to warming is not correlated to the degree of experimental warming [4] Additionally, the elevated air temperature we simulated (i.e. 0.72–0.94°C) was in line with the mean increase in atmospheric temperature during the past century (i.e. 0.74°C [1]) and the predicted temperature increases for the coming decades (i.e. 0.6–0.9°C [19]). This suggests that our simulation was highly relevant to the natural conditions of the experimental site. All in all, we found a species-specific response of plant senescence to warming in this study, suggesting that the current warming rates are exerting a profound influence on terrestrial ecosystems.

### Warming on plant senescence

As predicted, simulated warming did induce some significant changes in plant senescence (slowing down the decline in chlorophyll degradation and promoting higher N concentration in plant tissue under warmer conditions); however, these changes were found only in perennial species (Figure 2; Figure 3). During the senescence period of perennial species, the differences in chlorophyll and N concentration between warmed and control quadrates initially increased. This implied that the process of senescence, or degradation of chlorophyll, was slowed by warming. Thus, temperature seemed to play a modulating role during plant senescence. We found that leaf chlorophyll and N concentrations declined during late stage of senescence (the mechanism is not yet clear), causing similar levels in warmed and control treatments in the two perennial species. This trend was consistent with our prediction that temperature plays an important role in the process of senescence in opportunistic species that have riskier life strategies [3], [45]. This strategy reflects the tendency of the plant to gamble on the local environment of a particular habitat [42], [46]. Furthermore, our study did not find the variable effects of rising temperature on the cessation of growing season as previous studies reported [17], [47]. This inconsistency is probably due to the photoperiod, which primarily controls the termination of plant growth at the end of growing periods [47]. It is undeniable that the positive response of senescence process indicated that temperature can modulate a diverse set of community and ecosystem processes at high altitude regions even at the end of plant growth.

### Dynamics of N and chlorophyll during plant senescence

N status and chlorophyll levels, major factors of the senescence process, contribute to the enhancement of photosynthesis and work to offset the nutrient deficiencies [48]. In our study, large declines in N concentration during senescence paralleled with the loss of chlorophyll in the leaves of all four species from the control quadrates (Pearson's correlation coefficient R^2^ = 0.923, P<0.001). This occurred in all four species, and is supported by similar phenomenon found by Aerts et al. [42]. Additionally, we found higher chlorophyll levels and leaf N concentrations in *D. caeruleum* and *E. nutans* in warming quadrates during the plant senescence (Figure 2; Figure 3). This was a consequence of the chlorophyll biosynthesis (encompassing a series of enzymatic reactions) that was highly affected by temperature [49]. This finding implied that the capacity of photosynthesis was enhanced, although the evidence of photosynthetic parameters were lacking. Indeed, warming can result in an increase in photosynthesis capacity, due to an increase in chlorophyll and N concentrations [50], [51]. Moreover, leaf traits are more sensitive to warming than stem traits, which showed no significant changes in any species. Nevertheless, stem traits can also express plastic responses to temperature variations, responses that are particularly evident in nutrient resorption patterns [52], [53].

The levels of N concentration in senesced plant materials, similar to the N resorption proficiency [54], [55], were affected by warming treatment and may be species-specific. For example, Aerts et al. [43] found summer warming reduced senesced leaf N concentration both in *Betula* and *Rubus*; whereas, spring warming led to a reduction in only *Vaccinium*. In this study, few effects of elevated temperature were found on N concentration in senesced leaves and culms of all four species (Figure 3). Likewise, Norby et al. [42] did not find changes in leaf N content in either *Acer rubrum* or *Acer saccharum* after two years of warming treatment. In fact, to our knowledge, there is no current evidence to suggest that the ultimate nutrient concentration in litter is temperature dependent. Therefore, the underlying reasons for the different responses require further study. Moreover, it was clear that all leaves and culms of studied individuals in warmed quadrates completed the normal course of N resorption before the first killing frost. This ensured that N conservation was complete, and thus, presented a normal N concentration in senesced plant materials.

### Storage of TNC in roots

Partially consistent with our second hypothesis, we found that warming resulted in increased TNC storage in roots, but only in the *D. caeruleum* (Figure 4A). Warming improved plant growth and carbon assimilation when the temperature was maintained at the optimal degree for the plant species [49]. Therefore, warming-induced increases in the TNC of *D. caeruleum* roots may be a result of enhanced plant photosynthesis since non-structural carbohydrates are intermediate products of carbon assimilation. The increased accumulation of non-structural carbohydrate with warming corroborated the prediction set by modeling, which showed that an approximate 1% increase in tissue non-structural carbohydrate concentration for every 1°C rise [25]. In general, warming prompted the photosynthetic rate by enhancing the activities of photosynthetic enzymes. Our study suggests that, in response to warming, plants adopt a risky strategy, i.e. slowing the degradation of chlorophyll, to increase photosynthesis capacity and non-structural carbohydrate. Root TNC of *D. caeruleum* and *E. nutans* were much lower in spring, possibly due to the respiration during the winter. For example, Heilmeier et al. [29] reported that *Arctium tomentosum*, a biennial monocarp, uses a large proportion of winter carbohydrate stores (25% of their autumn biomass) for respiration. Warming-induced edge of TNC storage was still existed for *D. caeruleum* in the early spring of 2012, as it was inherited from the last season when storage represented an evolutionary relic in carbon allocation pattern [9].

Our study showed that autumn warming did not change the cessation of senescence, but enhanced the potential C uptake capacity that led to an increase in primary production. So far, the links between climate change and the terrestrial C cycle remain unclear. Warmer autumn seems to decrease the net ecosystem C uptake due to a larger increase in respiration rates [23]. While another study suggested that the net ecosystem C losses in response to autumn warming may not due to the decrease in plant C uptake, it may result from changes in the C budgets of other ecosystem compartments [30]. In any event, we did find a significant effect of warming on TNC storage. Given the species-specific responses of TNC storage to warming, changing community composition and structure as a result of warming should be considered in the near future. Indeed, many observations have clearly shown that climate warming will affect the structures of high-altitude communities in the medium- to long-term [56], [57].

### Differences in physiological responses among life forms

As expected, non-perennial species were more conservative and showed an inactive senescence responding to warming compared to perennial species. However, it is still unclear whether the differences between these life forms can be explained by their differential life history strategies. Annuals tend to adopt a higher relative growth rate (including senescence rate) compared to perennials, ensuring that annuals invest all available resources into flower and seed formation [29]. However, climate warming can lead to changes in the competitive balance between life forms by modifying their respective fitness due to differences in phenological sensitivity to temperature. These effects, therefore, may play a significant role in the distribution of vegetation and composition of grassland ecosystems. This also apply to other ecosystems, because global universality of importance of N resorption and TNC storage in autumn. Shifts in facilitation and competition mechanisms, in combination with the maintenance of high photosynthesis capacity, are expected to lead to lasting changes in ecosystem structure and function.

### Growth in 2012

Contrary to our expectations, we found little association between the level of TNC and individual above-ground biomass in our study. This illustrates that the warming treatment had no significant effect on the aboveground biomass production of any species during subsequent seasons. Similar findings were reported by Kleijn et al. [26], who showed a lack of competitive advantage from high TNC reserves in *V. album.* It may be that the increase of root TNC in *D. caeruleum* was insufficient to impact early growth during the next season. Previous defoliation or shoot-removal studies showed early growth effects occurred only with large changes in root TNC at the end of senescence [26], [58], [59]. In fact, a considerable fraction of TNC remains unused in a storage pool the following season [60]. In addition, many perennial plants store large quantities of TNC rather than use them directly for growth [26]. This stored resource may give plants a competitive advantage (for example an earlier start of spring-growth) or bridge temporal gaps that exist between resource availability and resource demand (for example during periods of drought, cold or calamities) [61]. These functions may be particularly relevant to alpine plant species grown under harsh conditions [47]. Aside from factors previously discussed, differences between treatments in the stored N may be another reasonable factor that impacts the plant growth [26], especially in alpine plants, which are not particularly limited by C [15]. In our study, it was not clear whether stored N in roots was significantly affected by experimental warming (data lacked), and so, it is difficult to attribute the lack of a growth response to the stored N.

The influence of autumn warming was not convolved with the phenological parameters of the following growing season, which implied that there was no direct contribution of TNC augment to the phenological events in the following seasons. To our knowledge, three different explanations may be possible. First, the onset of growing season depends on the cumulative temperature for many species, an effect not altered by experimental treatment or TNC accumulations. Second, the influence of TNC on further growth gradually subsided with the increased above-ground photosynthetic capacity after leaf unfolding [62], [63]. Finally, the magnitude of increased TNC was insufficient to influence the phenological timing parameters, especially in the alpine plants for which growth does not appear to be particularly limited by carbon status [15]. Overall, the association between non-structural carbohydrates and plants growth performance and phenological timing parameters needs further studies.

### Conclusions

In conclusion, our study has shown that autumn warming in an alpine meadow of the Tibetan Plateau can affect plant senescence processes by slowing chlorophyll degradation. Root TNC storage was subsequently increased as a result of the higher N concentration retention in leaves and culms. Importantly, the effects of warming on plant senescence differed among life forms, indicating perennial herbs were more susceptible to modifying their fitness to temperature than non-perennial species. This implies that warming will lead to changes in the competitive balance among life forms, which will lead to future shifts in vegetation distribution and species composition. Our results suggest that climate warming has already impacted, and will continue to affect, plant growth and carbon allocation in terrestrial ecosystems. Given the species-specific response of TNC accumulation among perennial species, these processes will be modulated by species composition and the relative abundance of climate-sensitive functional types in this region.
